# Whole-Genome Sequences of Two New *Caballeronia* Strains Isolated from Cryoturbated Peat Circles of the Permafrost-Affected Eastern European Tundra

**DOI:** 10.1128/MRA.00731-20

**Published:** 2020-07-30

**Authors:** Stefanie A. Hetz, Anja Poehlein, Marcus A. Horn

**Affiliations:** a Soil Microbiology, Institute of Microbiology, Leibniz University Hannover, Hannover, Germany; b Department of Genomic and Applied Microbiology, Institute of Microbiology and Genetics, Georg August University of Göttingen, Göttingen, Germany; University of Southern California

## Abstract

Annotated genomes of *Caballeronia* strains SBC1 and SBC2 from acidic permafrost suggest a new species with a facultative lifestyle via oxygen and nitrate respiration. Thus, a contribution to nitrogen cycling in cold and low-pH environments is anticipated.

## ANNOUNCEMENT

Cryoturbated peat circles (PCs) (62°57′E, 67°03′N) contain up to 2 mM pore water nitrate, emit large amounts of nitrous oxide (1, 2), and host new nitrate reducers (3). SBC1 and SBC2 were isolated from serial PC sediment (pH 4.2) dilutions by plating on semisolid modified R2A medium (1:10 diluted DSMZ 830 medium, 0.5% [wt/vol] K_2_HPO_4_, 7 g liter^−1^ Gelrite [pH 6]) and incubating for 7 days at 15°C. Single colonies were picked and purified by restreaking four times onto the same medium.

High-molecular-weight DNA (HWD) for Nanopore sequencing was isolated with the MasterPure complete DNA and RNA purification kit (Biozym, Hessisch Oldendorf, Germany) from cells grown in liquid modified R2A medium (pH 5). HWD quality was checked on a Bioanalyzer 2100 using the DNA 12000 kit (Agilent Technologies, Waldbronn, Germany), and HWD was quantified with the Qubit double-stranded DNA (dsDNA) high-sensitivity (HS) assay kit (Life Technologies GmbH, Darmstadt, Germany); 1.5 μg HWD was used for library preparation employing the ligation sequencing kit 1D (SQK-LSK109) and the native barcode expansion kit (EXP-NBD114, barcode 15; Oxford Nanopore Technologies, Oxford, UK). Sequencing was performed for 72 h on the MinION Mk1B system with a SpotON flow cell R9.4.1 using MinKNOW v19.10.1, with Guppy v3.3.3 for base calling and demultiplexing. Totals of 67,997 reads with an average length of 4,873 bp (*N*_50_, 27,443 bp) for SBC1 and 330,181 reads with an average length of 9,102 bp (*N*_50_, 15,651 bp) for SBC2 were obtained.

Genomic DNA for Illumina shotgun sequencing was isolated via PCI extraction (4) and checked via spectrophotometry (DS-11; DeNovix, Inc., Wilmington, DE, USA). Illumina shotgun libraries were prepared using the Nextera XT DNA sample preparation kit, sequenced on a MiSeq system using reagent kit v3 with 600 cycles (2 × 300 bases; Illumina, San Diego, CA, USA), and resulted in totals of 3,249,515 (SBC1) and 2,281,633 (SBC2) paired-end reads per strain. Illumina reads were quality filtered using Trimmomatic v0.39 (5). Unicycler v0.4.6 (6) was used to perform a hybrid assembly, resulting in a closed circular chromosome (4,010,354 bp) and 4 closed plasmids (280,710 to 1,996,666 bp) for SBC1 and a closed circular chromosome (3,989,243 bp) and 7 closed plasmids (120,676 to 1,990,521 bp) for SBC2, as validated using Bandage v2.1 (7). Coverage was determined using Qualimap v2.2.1 (8) by mapping Illumina and Nanopore reads on the closed genomes using Bowtie 2 v2.3.5.1 (9) and minimap2 (10), respectively. Coverages for SBC1 and SBC2 were 98.4× and 62.4× (Illumina) and 62.8× and 162.8× (Nanopore), respectively. The overall GC contents (BioEdit v7.0.5.3 [11]) of SBC1 and SBC2 were 59.69% and 59.52%, respectively. Annotation with Prokka v1.14.0 (12) revealed the presence of 5 rRNA operons for both genomes, with 8,050 and 8,520 predicted protein-encoding genes and 59 and 60 tRNA genes for SBC1 and SBC2, respectively. Default parameters were used for all software.

**FIG 1:**
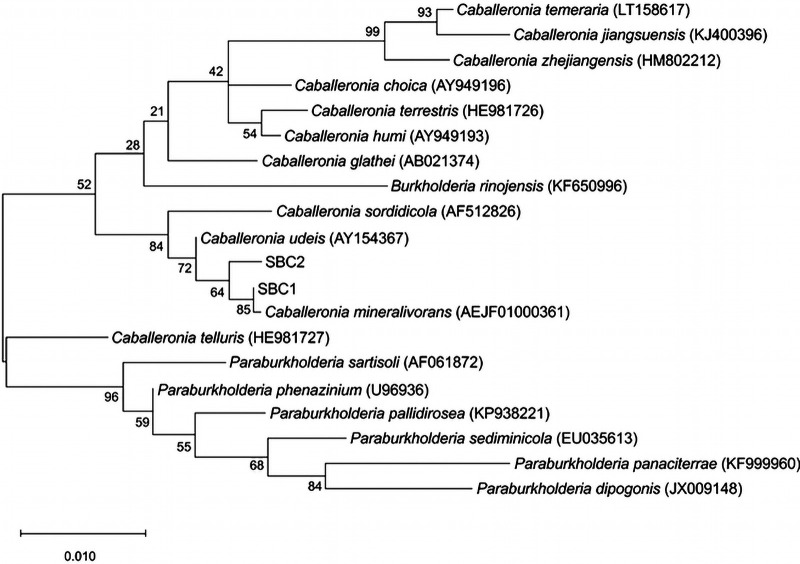
Maximum composite likelihood tree of 16S rRNA genes aligned with MUSCLE (17) and rooted by midpoint rooting. Branches are scaled in terms of the expected number of substitutions per site. The percentage of trees in which the associated taxa clustered together is shown next to the branches. Initial trees for the heuristic search were obtained automatically by applying the neighbor-joining and BioNJ algorithms to a matrix of pairwise distances estimated using the maximum composite likelihood and the Tamura-Nei model (18) and then selecting the topology with a superior log likelihood value. The closest relative of SBC1 and SBC2 was *C. mineralivorans* from a fungal ectomycorrhizosphere in acidic and nutrient-poor forest soil (19). Tree construction was conducted with MEGA X, and GenBank accession numbers of 16S rRNA gene sequences are provided in parentheses next to species names (20).

SBC1 and SBC2 were affiliated with Caballeronia mineralivorans (Fig. 1); 53 to 57% of SBC1 and SBC2 genomes aligned with the *C. mineralivorans* genome, and the average nucleotide identity using the MUMmer algorithm (ANIm) (JSpeciesWS [13]) was 88.3%. The ANIm of SBC1 compared to SBC2 was 99.7%, suggesting that SBC1 and SBC2 represent a new species of the genus *Caballeronia* (14, 15). SBC1 and SBC2 encode multiple nitrate reductases of the *narG*, *napA*, and *nasA* types, as well as nitrite (*nirBD*) and nitric oxide (*norV*) reductases (Pathway Tools v23.0 [16]).

### Data availability.

These whole-genome shotgun projects have been deposited in DDBJ/ENA/GenBank under the accession numbers CP049156.1 (chromosome) and CP049157.1 to CP049160.1 (plasmids) for SBC1 and under the accession numbers CP049316.1 (chromosome) and CP049317.1 to CP049323.1 (plasmids) for SBC2. BioProject accession numbers for SBC1 and SBC2 are PRJNA604524 and PRJNA604525, and SRA accession numbers are SRP250914 and SRP250916, respectively.
